# Supplementary figures and images for: Using transplantation to restore seagrass meadows in a protected South African lagoon
Source: PeerJ. 2023 Nov 29;11:e16500. doi: 10.7717/peerj.16500 (PMC10693235; doi:10.7717/peerj.16500)

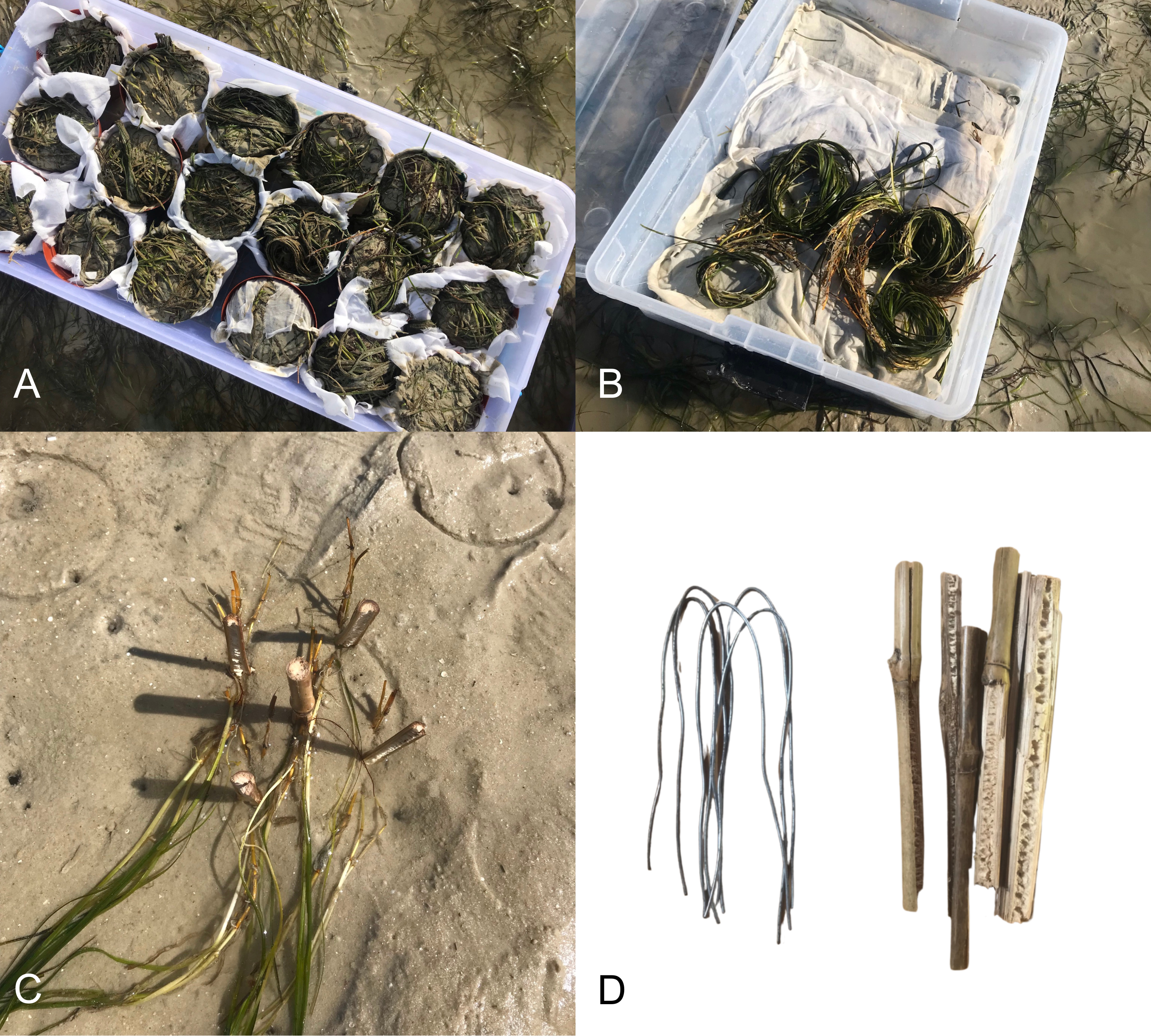

Supplement: Supplemental Information 1 — (A) Collected 10 cm diameter cores; (B) Collected seagrass shoots; (C) Three shoots gathered per peg, with five pegs per core; and (D) Metal pegs and bamboo pegs used to anchor seagrass shoots. [file peerj-11-16500-s001.png]

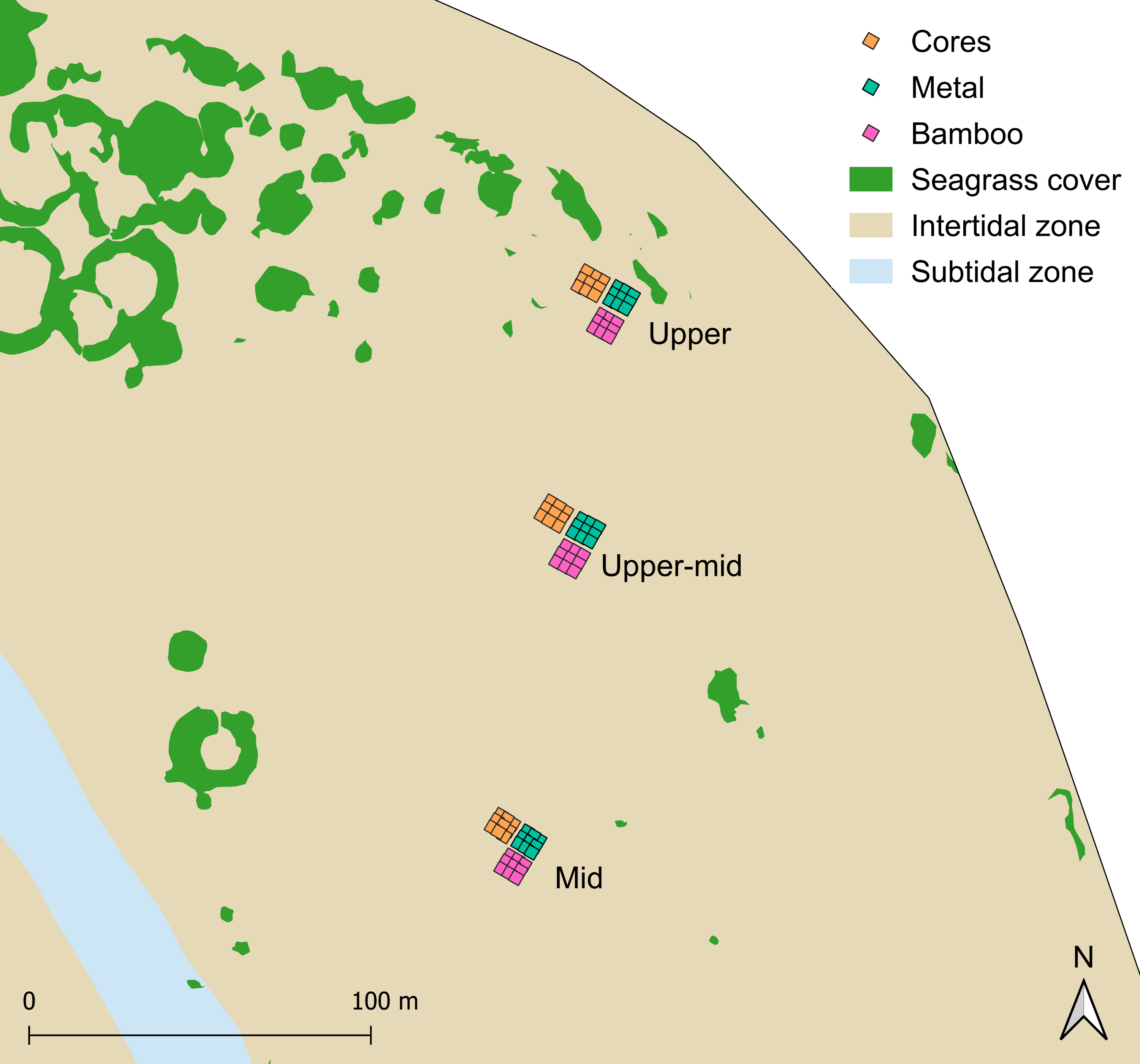

Supplement: Supplemental Information 2 — Transplant plots across sites used cores (pink boxes), shoots anchored by metal pegs (green boxes), and shoots anchored by bamboo pegs (orange boxes), across the tidal gradient using three replicated planting patterns. [file peerj-11-16500-s002.png]

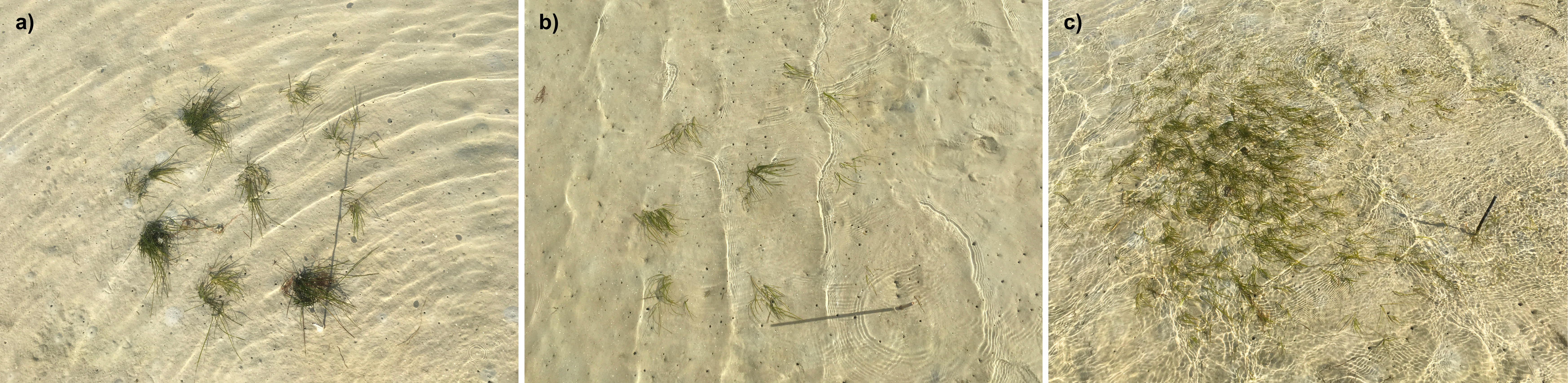

Supplement: Supplemental Information 3 — (a) initial monitoring at 6 days post-transplantation, (b) 83 days post-transplantation, showing the initial decrease in cover, and (c) after 335 days post-transplantation, whereby transplanted seagrass increased to form a complete patch. Photo credit: Katie M. Watson. [file peerj-11-16500-s003.png]
