# Supplementary material for: Using transplantation to restore seagrass meadows in a protected South African lagoon
Source: PeerJ. 2023 Nov 29;11:e16500. doi: 10.7717/peerj.16500 (PMC10693235; doi:10.7717/peerj.16500)
Supplement: Supplemental Information 4 — The fixed effect of the model is made by the interaction between planting pattern (“Line”, “Dense”, and “Bullseye”), planting material (“Cores”, shoots anchored with “Metal” or “Bamboo” pegs), site (“Upper”, “Upper-mid”, and “Mid”) and the number of “Sandprawn holes”, with the addition of “Epiphytes” percentage cover. “Days since transplantation” and “Transplant plot” were included as random effects. The intercept coefficient refers to shoots anchored with “Bamboo” pegs, planted in a “Line” pattern, in the “Upper” intertidal planting site with both a high number of sandprawn holes and high epiphyte cover. Intercept has negative relationship with survival, ∴relationship between coefficients and factors are inverse. Asterisks indicate positive significant effects on transplant survival despite negative coefficients due to lower numbers of sandprawn holes, and significant effects without asterisks indicate negative effects on transplant plot survival despite positive coefficients due to high numbers of sandprawn holes [file peerj-11-16500-s004.docx]

**Table S1.** **Results from the best generalized linear mixed models fit for transplant plot survival (%).**

The log coefficients of fixed effects are reported with standard error (**±** SE) in brackets, and significant effects highlighted in bold. The fixed effect of the model is made by the interaction between planting pattern (“Line”, “Dense”, and “Bullseye”), planting material (“Cores”, shoots anchored with “Metal” or “Bamboo” pegs), site (“Upper”, “Upper-mid”, and “Mid”) and the number of “Sandprawn holes”, with the addition of “Epiphytes” percentage cover. “Days since transplantation” and “Transplant plot” were included as random effects. The intercept coefficient refers to shoots anchored with “Bamboo” pegs, planted in a “Line” pattern, in the “Upper” intertidal planting site with both a high number of sandprawn holes and high epiphyte cover. Intercept has negative relationship with survival, ∴ relationship between coefficients and factors are inverse. Asterisks indicate positive significant effects on transplant survival despite negative coefficients due to lower numbers of sandprawn holes, and significant effects without asterisks indicate negative effects on transplant plot survival despite positive coefficients due to high numbers of sandprawn holes.

| **Formula**: Survival (log + 1) ~ Planting pattern * Material * Site * Sandprawn holes + Epiphytes + (1\|Days since transplantation) + (1\|Transplant plot)  **AIC**: 18794.16 | | |
| --- | --- | --- |
| **Effect** | **Coefficient (± SE)** | ***p*** |
| **Intercept** | **11.335 (9.09)** | **< 0.001** |
| Planting pattern(Dense) | 1.409 (12.16) | 0.16 |
| Planting pattern(Bullseye) | 1.309 (12,37) | 0.19 |
| **Material(Cores)** | **-3.465 (12.78)** | **< 0.001** |
| **Material(Metal)** | **-3.398 (12.28)** | **< 0.001** |
| Site(Upper-mid) | 1.342 (12.32) | 0.18 |
| Site(Mid) | 1.042 (12.50) | 0.30 |
| **Sandprawn holes*** | **-2.379 (0.03)** | **0.02** |
| **Epiphytes** | **8.006 (0.02)** | **< 0.001** |
| Planting pattern(Dense):Material(Cores) | -0.187 (17.69) | 0.85 |
| Planting pattern(Bullseye):Material(Cores) | -0.773 (17.64) | 0.44 |
| Planting pattern(Dense):Material(Metal) | 0.863 (16.56) | 0.39 |
| Planting pattern(Bullseye):Material(Metal) | -1.305 (17.80) | 0.19 |
| Planting pattern(Dense):Site(Upper-mid) | -1.235 (16.73) | 0.22 |
| Planting pattern(Bullseye):Site(Upper-mid) | -0.964 (16.95) | 0.34 |
| Planting pattern(Dense):Site(Mid) | 0.076 (17.42) | 0.94 |
| Planting pattern(Bullseye):Site(Mid) | -0.010 (17.26) | 0.99 |
| Material(Cores):Site(Upper-mid) | 0.278 (17.39) | 0.78 |
| Material(Metal):Site(Upper-mid) | 1.529 (17.27) | 0.13 |
| Material(Cores):Site(Mid) | 1.513 (17.37) | 0.13 |
| Material(Metal):Site(Mid) | 0.804 (17.47) | 0.42 |
| Planting pattern(Dense):Sandprawn holes | -1.534 (0.05) | 0.16 |
| Planting pattern(Bullseye):Sandprawn holes | -1.645 (0.05) | 0.10 |
| **Material(Cores):Sandprawn holes** | **3.493 (0.04)** | **< 0.001** |
| Material(Metal):Sandprawn holes | 1.488 (0.05) | 0.14 |
| **Site(Upper-mid):Sandprawn holes*** | **-3.663 (0.04)** | **< 0.001** |
| **Site(Mid):Sandprawn holes*** | **-2.796 (0.04)** | **0.01** |
| Planting pattern(Dense):Material(Cores):Site(Upper-mid) | 0.210 (23.70) | 0.83 |
| Planting pattern(Bullseye):Material(Cores):Site(Upper-mid) | 0.225 (23.91) | 0.82 |
| Planting pattern(Dense):Material(Metal):Site(Upper-mid) | -0.152 (23.36) | 0.88 |
| Planting pattern(Bullseye):Material(Metal):Site(Upper-mid) | 1.387 (24.32) | 0.17 |
| Planting pattern(Dense):Material(Cores):Site(Mid) | -0.808 (24.21) | 0.42 |
| Planting pattern(Bullseye):Material(Cores):Site(Mid) | -0.360 (23.95) | 0.72 |
| Planting pattern(Dense):Material(Metal):Site(Mid) | -0.724 (23.87) | 0.47 |
| Planting pattern(Bullseye):Material(Metal):Site(Mid) | 1.434 (24.60) | 0.15 |
| Planting pattern(Dense):Material(Cores):Sandprawn holes | 0.815 (0.07) | 0.42 |
| **Planting pattern(Bullseye):Material(Cores):Sandprawn holes** | **2.336 (0.06)** | **0.02** |
| Planting pattern(Dense):Material(Metal):Sandprawn holes | -0.500 (0.06) | 0.62 |
| **Planting pattern(Bullseye):Material(Metal):Sandprawn holes** | **2.714 (0.07)** | **0.01** |
| Planting pattern(Dense):Site(Upper-mid):Sandprawn holes | 1.504 (0.06) | 0.13 |
| Planting pattern(Bullseye):Site(Upper-mid):Sandprawn holes | 1.587 (0.06) | 0.11 |
| Planting pattern(Dense):Site(Mid):Sandprawn holes | 0.209 (0.06) | 0.83 |
| Planting.pattern(Bullseye):Site(Mid):Sandprawn holes | -0.104 (0.06) | 0.92 |
| Material(Cores):Site(Upper-mid):Sandprawn holes | 0.207 (0.06) | 0.84 |
| Material(Metal):Site(Upper-mid):Sandprawn holes | -0.228 (0.06) | 0.82 |
| Material(Cores):Site(Mid):Sandprawn holes | -0.831 (0.05) | 0.41 |
| Material(Metal):Site(Mid):Sandprawn holes | 0.337 (0.06) | 0.74 |
| Planting pattern(Dense):Material(Cores):Site(Upper-mid):Sandprawn holes | -0.513 (0.08) | 0.61 |
| **Planting pattern(Bullseye):Material(Cores):Site(Upper-mid):Sandprawn holes*** | **-1.995 (0.07)** | **0.05** |
| Planting pattern(Dense):Material(Metal)Site(Upper-mid):Sandprawn holes | -0.028 (0.08) | 0.98 |
| **Planting pattern(Bullseye):Material(Metal):Site(Upper-mid):Sandprawn holes*** | **-2.981 (0.08)** | **< 0.001** |
| Planting pattern(Dense):Material(Cores):Site(Mid):Sandprawn holes | 0.512 (0.08) | 0.61 |
| Planting pattern(Bullseye):Material(Cores):Site(Mid):Sandprawn holes | -0.905 (0.08) | 0.37 |
| Planting pattern(Dense):Material(Metal):Site(Mid):Sandprawn holes | 0.157 (0.08) | 0.88 |
| **Planting pattern(Bullseye):Material(Metal):Site(Mid):Sandprawn holes*** | **-2.513 (0.08)** | **0.01** |
