# Supplementary material for: Using transplantation to restore seagrass meadows in a protected South African lagoon
Source: PeerJ. 2023 Nov 29;11:e16500. doi: 10.7717/peerj.16500 (PMC10693235; doi:10.7717/peerj.16500)
Supplement: Supplemental Information 6 — The log coefficients of fixed effects are reported with standard error (± SE) in brackets, and significant effects highlighted in bold. The fixed effect of the model is made by the effect of planting material (“Cores”, shoots anchored with “Metal” or “Bamboo” pegs). “Days since transplantation” and “Transplant plot” were included as random effects. The intercept coefficient refers to “Cores”. [file peerj-11-16500-s006.docx]

**Table S3. Results from the best generalized linear mixed models fit for Shannon-Wiener diversity index.**

The log coefficients of fixed effects are reported with standard error (**±** SE) in brackets, and significant effects highlighted in bold. The fixed effect of the model is made by the effect of planting material (“Cores”, shoots anchored with “Metal” or “Bamboo” pegs). “Days since transplantation” and “Transplant plot” were included as random effects. The intercept coefficient refers to “Cores”.

| **Formula**: Shannon Diversity Index (log + 1) ~ Material + (1\|Days since transplantation) + (1\|Transplant plot)  **AIC**: -1590.57 | | |
| --- | --- | --- |
| **Effect** | **Coefficient (± SE)** | ***p*** |
| **Intercept** | **31.230 (0.02)** | **< 0.001** |
| **Material(Metal)** | **-3.657 (0.02)** | **< 0.001** |
| **Material(Bamboo)** | **-4.985 (0.02)** | **< 0.001** |
